# A Possible Link Between Cutaneous Melanoma and Uveal Melanoma: A Multicenter Retrospective Cohort Study

**DOI:** 10.3390/cancers18172826

**Published:** 2026-09-01

**Authors:** Maya Eiger-Moscovich, Natan Lishinsky-Fischer, Shahar Frenkel, Jacob Pe’er, Amit Moscovich, Zvi Gur

**Affiliations:** 1 Division of Ophthalmology, Hadassah Medical Center, Jerusalem 9112001, Israel; shahar.frenkel@mail.huji.ac.il (S.F.); peer@hadassah.org.il (J.P.); zvigur@hadassah.org.il (Z.G.); 2 Faculty of Medicine, Hebrew University, Jerusalem 9112102, Israel; natan.lishinsky@mail.huji.ac.il; 3 Department of Statistics and Operations Research, Tel Aviv University, Tel Aviv 6997801, Israel; mosco@tauex.tau.ac.il

**Keywords:** uveal melanoma, cutaneous melanoma, TriNetX

## Abstract

Purpose: Cutaneous melanoma (CM) and uveal melanoma (UM) derive from melanocytes. Nevertheless, they differ in genetic drivers and progression pathways. Previous research examining the relationship between CM and UM has been constrained by small cohorts and the rarity of UM. The aim of this study was to determine whether UM occurs more frequently in patients with a CM compared to matched patients with keratinocyte carcinoma (KC, squamous or basal cell carcinoma of the skin). Patients with KC were selected as the control to account for the excess risk due to sun exposure and access to healthcare. Methods: We performed a large, multicenter retrospective cohort study using TriNetX, a global health research network and real-world data platform. Cohort 1 had CM or melanoma in situ (MIS) and no history of KC. Cohort 2 had KC and no history of CM or MIS. The main outcome was the incidence of malignant neoplasms of the choroid or ciliary body over a 5-year follow-up, including diagnoses recorded before the index skin cancer. Patients who had metastatic disease were excluded to remove potential misidentifications of choroidal metastasis as UM. A 1:1 propensity score matching (PSM) was used to match demographic and clinical characteristics. Odds ratios (ORs) with 95% confidence intervals (CIs) and corresponding two-sided *p*-values were used to compare categorical outcomes. Ophthalmologic follow-up was evaluated to rule out surveillance bias. Results: The CM cohort included 57,352 patients. The KC cohort included 137,083 patients. After PSM each cohort included 52,860 patients. UM occurred in 53 CM patients (0.1%) compared with 13 KC patients (0.02%). This corresponds to an OR of 4.08 (95% CI [2.22, 7.49]; *p* < 0.001). UM diagnosis preceded CM in 70% of patients with both diagnoses. The association persisted in a sensitivity analysis after excluding patients with UM documented before CM (HR 14.32; 95% CI 7.96–25.76). Conclusions: Cutaneous melanoma is associated with a significantly higher risk of UM compared to KC. These findings suggest a link between cutaneous and uveal melanoma and may offer a possible role for ophthalmic surveillance in CM patients and dermatology surveillance in UM patients.

## Simple Summary

The aim of this study was to determine whether there is a link between the development of cutaneous melanoma and uveal melanoma, unrelated to sun exposure. In a large, multicenter retrospective cohort study using TriNetX, a global health research network and real-world data platform, we found that cutaneous melanoma is associated with a significantly higher risk of uveal melanoma compared to keratinocyte carcinoma (squamous or basal cell carcinoma of the skin). These findings suggest a link between cutaneous and uveal melanoma and may offer a possible role for ophthalmic surveillance in cutaneous melanoma patients and dermatology surveillance in uveal melanoma patients.

## 1. Introduction

Uveal melanoma (UM) is the most common primary intraocular malignancy in adults. Nevertheless, it is a rare disease, with an incidence of approximately 5–7 cases per million people per year [1,2,3]. Risk factors include fair skin, light-colored eyes, congenital ocular melanocytosis, and the *BAP1* tumor predisposition syndrome [1]. UM arises from melanocytes of the iris, ciliary body, or choroid, with the choroid accounting for about 85–90% of cases, the ciliary body approximately 6%, and the iris about 4% [1,2,4]. Eye-preserving treatments, including plaque brachytherapy and proton beam radiation, are the mainstay of treatment, with enucleation only performed in very advanced cases [1,2,5]. Despite effective local control, up to 50% of patients ultimately develop metastases, most commonly to the liver. Once metastases occur, survival remains poor, although the development of new treatments such as the bispecific T-cell engager tebentafusp, the PKC inhibitor darovasertib, and percutaneous hepatic perfusion with melphalan show potential for improved survival [1,6,7,8,9].

Cutaneous melanoma (CM) is a much more prevalent tumor, with 40 cases per million people per year, and incidence increasing steadily over recent decades. Despite representing about 4% of skin cancers, it accounts for up to 75% of skin cancer-related deaths [10]. Risk factors include fair skin, light-colored eyes, ultraviolet radiation (UV), genetic susceptibility, numerous or dysplastic nevi, and immunosuppression [11]. Early-stage melanoma is primarily managed with surgical excision, while advanced or metastatic melanoma is treated with immune checkpoint inhibitors (anti-PD-1 and anti-CTLA-4 antibodies) and targeted therapies for tumors harboring *BRAF* or related mutations [12,13]. Prognosis is excellent when melanoma is detected early but declines markedly with regional or distant metastasis [14].

CM and UM derive from neural crest melanocytes that migrated to the epidermis or the eye. Nevertheless, they differ in genetic drivers and progression pathways. In a review of 84,836 melanoma cases diagnosed between 1985 and 1994 by the National Cancer Database, the percentage of cutaneous melanomas was 92%, ocular melanomas comprised 5%, mucosal melanomas 1% and 2% melanomas of unknown primary [15]. Among the ocular melanomas, 84% arise from the uvea, 6% from the conjunctiva, and 10% from the eyelids and orbit [15].

Previous research examining the relationship between CM and UM has been constrained by small cohorts, limited follow-up, and single-center designs, which limit statistical power in studying such a rare malignancy [16,17,18,19]. Given the low incidence of UM, population-based, multicenter resources are essential to provide robust evidence for potential epidemiologic links between these melanocytic tumors. The TriNetX global health research network aggregates de-identified health records from diverse healthcare organizations worldwide, offering the statistical strength and geographic breadth needed to investigate rare associations in real-world clinical settings.

The aim of this study was to determine whether patients with a history of CM have a higher risk of developing UM compared with matched patients with keratinocyte carcinoma (KC, squamous or basal cell carcinoma of the skin). In designing this study, patients with KC were selected as the control cohort because these malignancies share key environmental risk factors with CM, most notably chronic sun exposure. This choice is used to evaluate the excess risk of developing both CM and UM beyond that seen with other UV-related skin cancers. Patients who had metastatic disease were excluded from both groups, in an attempt to avoid misclassification of metastatic CM to the choroid as uveal melanoma.

## 2. Materials and Methods

### 2.1. Study Design

We conducted a retrospective cohort study using the TriNetX global collaborative network, a federated health research platform that, as of August 2025, contains de-identified electronic health records (EHRs) from more than 180 million patients across over 150 healthcare organizations worldwide, primarily in North America and Europe. The platform provides standardized access to demographic information, diagnoses, procedures, medications, laboratory results, and healthcare utilization metrics.

Study variables were identified using the International Classification of Diseases, 10th Revision, Clinical Modification (ICD-10-CM) and International Classification of Diseases for Oncology, 3rd Edition (ICD-O-3). Procedures were identified using Current Procedural Terminology (CPT) codes. Medications were captured using RxNorm, the Anatomical Therapeutic Chemical (ATC) classification system, and the Healthcare Common Procedure Coding System (HCPCS). Laboratory results were retrieved using the Logical Observation Identifier Names and Codes (LOINC) system.

### 2.2. Inclusion and Exclusion Criteria

Using the TriNetX codes-query system, we constructed two cohorts of patients:

Cohort 1 (CM): Patients who had a diagnosis consistent with melanoma, identified through ICD-10-CM or ICD-O-3 codes for malignant melanoma of skin (C43) or melanoma in situ (D03), who had undergone skin biopsy (CPT 11102, 11103, 11104, 11105, 11106, 11107, 11600, 11601, 11602, 11603, 11604, 11606, 11300, 11301, 11302, 11303). Patients with KC were excluded.

Cohort 2 (KC): Patients who had a diagnosis of keratinocyte carcinoma (KC, squamous or basal cell carcinoma of the skin) as identified by ICD-10-CM code C44 or ICD-O-3 codes 8090/3, 8070/3, 805-808, and who had undergone skin biopsy. CM patients were excluded.

In both cohorts, the requirement of recorded skin biopsy prior to the index event was added in order to minimize the potential for misclassification, thereby increasing the specificity of the identified skin cancer diagnoses.

In both cohorts, patients who had metastatic disease, identified through ICD-10-CM or ICD-O-3 codes for secondary malignant neoplasm (ICD-10-CM C76-80), were excluded.

**Figure 1:**
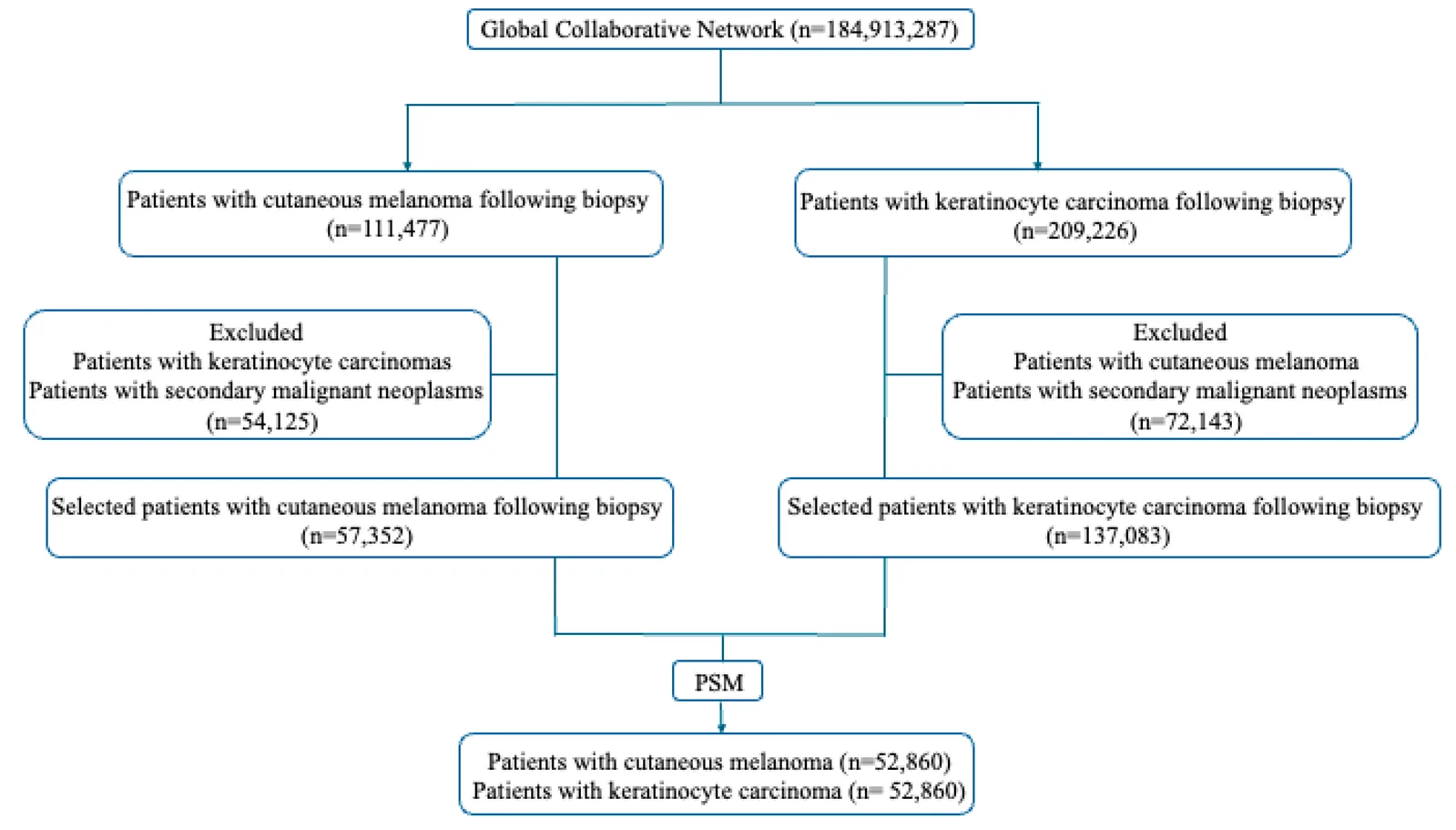
Flowchart of the cohort selection process from the TriNetX global electronic health record network.

Figure 1 illustrates the cohort selection process.

The index date was defined as the date of the first qualifying diagnosis of CM (or KC in the comparator cohort) after fulfillment of all eligibility criteria. Follow-up for the development of malignant neoplasms of the choroid or ciliary body began on the index date and continued for up to 5 years. The primary analysis evaluated the association between CM and UM and was not intended to estimate the future risk of developing UM following a CM diagnosis. Because patients with previously documented UM were not excluded, the analysis included both prevalent and incident UM cases.

### 2.3. Propensity Score Matching

Propensity score matching (PSM) was conducted using a broad set of demographic, clinical, and healthcare utilization variables. All covariates included in the propensity score model, including treatment-related variables, were assessed prior to the index date to ensure that only baseline characteristics were considered and to minimize potential bias from post-index exposures. Matching was conducted using a 1:1 nearest-neighbor algorithm with a caliper width of 0.1 of the pooled standard deviation of the logit of the propensity score. Demographic factors included age at index, sex (female or male), race and ethnicity (White, Black or African American, and Hispanic or Latino), as well as vital status (deceased).

Comorbidity variables encompassed overweight or obesity, nicotine dependence, hypertensive diseases, disorders of lipoprotein metabolism and other lipidemias, diabetes mellitus, and chronic kidney disease. Ocular comorbidities included diseases of the eye and adnexa, age-related cataract, glaucoma, and other retinal disorders. Cancer-related covariates included secondary malignant neoplasms of other and unspecified sites, lymph nodes, and respiratory or digestive organs.

Healthcare utilization factors comprised ambulatory visits, emergency visits, and encounters for examination of the eyes and vision. In addition, detailed ophthalmologic service variables were included, capturing medical examination and evaluation visits (comprehensive or intermediate; established or new patients). Factors influencing health status and contact with health services (ICD-10-CM codes Z00-Z99) were also included as covariates. This category encompasses health examinations, genetic susceptibility, preventive and reproductive healthcare encounters, socioeconomic and psychosocial circumstances, body mass index, lifestyle-related factors, and personal or family history and other conditions influencing health status. Finally, treatment-related variables included receipt of chemotherapy, corticosteroids, antimetabolites, and alkylating agents.

### 2.4. Outcomes

To assess the occurrence of UM, we evaluated the development of malignant neoplasms of the choroid or ciliary body (ICD-10-CM and ICD-O C69.3 and C69.4) over a 5-year follow-up, including patients with malignant neoplasms of the choroid or ciliary body prior to development of skin cancer. Malignant neoplasms of the iris were not included since there is no matching ICD-10 code. Ophthalmologic follow-up was compared between the cohorts to assess surveillance bias. To assess residual confounding and the robustness of the matched cohorts, we used appendicitis, inguinal hernia, carpal tunnel release, arthroscopy of the knee, Bell’s palsy and death as negative control outcomes.

### 2.5. Statistical Analysis

Odds ratios (ORs) with 95% confidence intervals (CIs) were estimated for outcome of interest as follows:

$$OR=\frac{Cohort\;1\;with\;outcome}{Cohort\;1\;without\;outcome}/\frac{Cohort\;2\;with\;outcome}{Cohort\;2\;without\;outcome}$$

Two-sided *p*-values for ORs were calculated using the Wald test based on the natural logarithm of the OR and its standard error, with statistical significance defined as *p* < 0.05. All analyses were performed on 1:1 propensity score-matched cohorts. Because potential confounding was addressed during the matching process, no further covariate adjustment was applied, leaving cohort assignment as the principal factor differentiating the groups.

Time-to-event analyses were performed using Kaplan–Meier survival curves to evaluate the cumulative incidence of the outcome over the follow-up period. Differences between cohorts were assessed using the log-rank test, with statistical significance defined as a two-sided *p*-value < 0.05. Hazard ratios (HRs) with 95% CIs were calculated using Cox proportional hazards regression. The proportional hazards assumption was assessed, with a *p*-value < 0.01 considered indicative of a violation of the assumption. When the proportional hazards assumption was not met, the KM curves and log-rank test were used as the primary assessment of differences in outcome occurrence over time, and HR estimates were interpreted with caution.

### 2.6. Ethical Statement

TriNetX complies with data privacy regulations applicable to participating healthcare organizations (HCOs), including the Health Insurance Portability and Accountability Act (HIPAA) in the United States, the General Data Protection Regulation (GDPR) in Europe, the Lei Geral de Proteção de Dados (LGPD) in Brazil, and other relevant regional regulations. Patient data are de-identified using processes that have been formally validated by qualified experts in accordance with Section §164.514(b)(1) of the HIPAA Privacy Rule. In addition, TriNetX provides technical support for the integration and onboarding of HCO data into the platform. The Institutional Review Board of the Hadassah Medical Center waived the requirement for ethics approval.

## 3. Results

**Figure 2:**
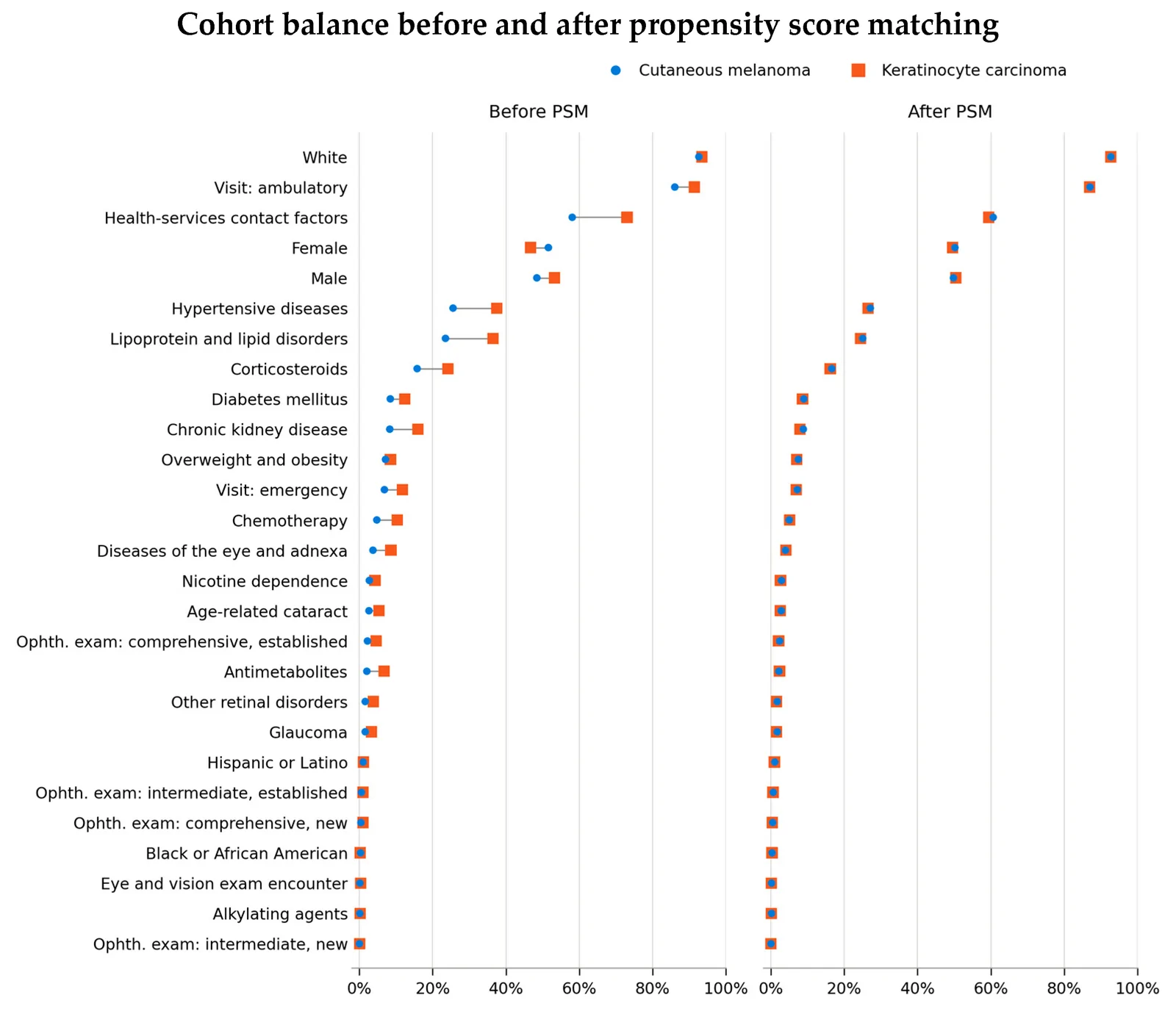
Prevalence of each binary characteristic in the two cohorts, before and after 1:1 propensity score matching (PSM). Paired points connected by a line show the two cohorts for a single characteristic in percentage points. Before matching, keratinocyte carcinoma patients had a higher prevalence for every characteristic except female sex. After matching, the paired points coincide throughout.

Before propensity score matching, the study population included 57,352 patients with cutaneous melanoma and 137,083 patients with keratinocyte carcinomas. Patients with melanoma were, on average, younger at diagnosis (61.39 ± 15.32 years vs. 68.8 ± 12.43 years, [mean ± stdev]), and more frequently female (51.57% vs. 46.75%) compared with the keratinocyte carcinoma cohort. CM patients had a lower prevalence of comorbidities such as hypertension, diabetes, dyslipidemia, obesity and nicotine dependence. KC patients had more unrelated eye diseases, including cataract, glaucoma and retinal disorders. After 1:1 PSM, both groups were balanced, with 52,860 patients in each cohort, and baseline characteristics were well-matched across demographics, comorbidities, ocular conditions, and healthcare utilization variables. The characteristics of both cohorts, before and after PSM, are presented in Table 1 and Figure 2.

The duration of follow-up was comparable between cohorts, with a mean follow-up of 1062.9 days in the CM cohort and 1066.5 days in the KC cohort. The median follow-up was identical in both cohorts (1105 days), with similar variability (interquartile range: 1440 vs. 1400 days).

**Figure 3:**
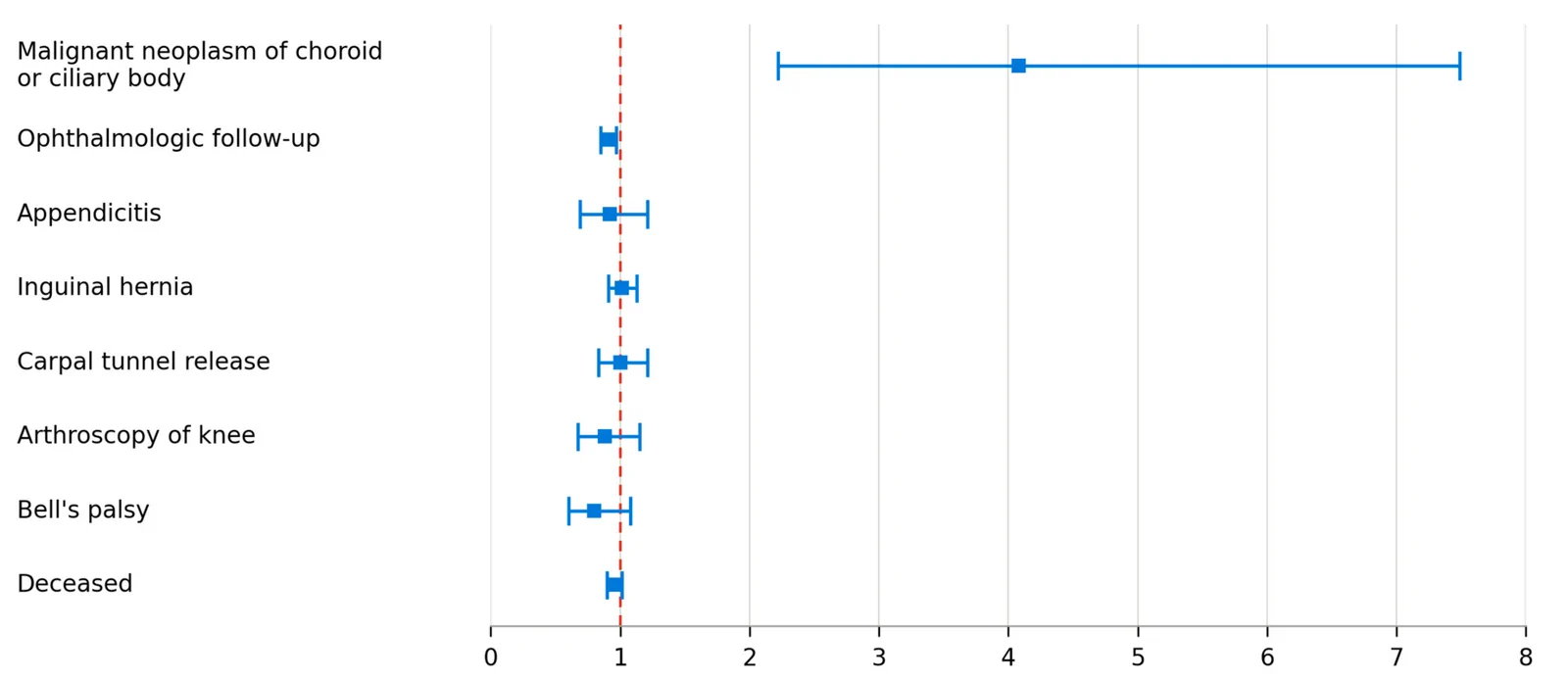
Odds ratios with 95% confidence intervals for each outcome over 5 years of follow-up, CM/KC (cutaneous melanoma case number divided by keratinocyte carcinoma in PSM-matched cases). An odds ratio above 1 indicates higher odds in the cutaneous melanoma cohort. See Table 2 for the raw data.

Following matching, we evaluated the development of UM. Malignant neoplasms of the choroid or ciliary body occurred in 53 CM patients (0.1%) compared with 13 KC patients (0.02%). This corresponds to an OR of 4.08 (95% CI [2.22, 7.49]; *p* < 0.001). These results are presented in Table 2 and Figure 3. Assessing the timeline, 37 patients developed UM prior to CM (70%), while only 16 patients developed UM after CM diagnosis (30%), indicating that the observed association was predominantly driven by patients in whom UM had been diagnosed before CM.

Ophthalmologic follow-up, included as a control variable to assess surveillance bias, was slightly less frequent among patients with CM (3.11% vs. 3.41%; OR 0.91; 95% CI [0.85, 0.97]; *p* = 0.005). In contrast, the negative control outcomes, including appendicitis, inguinal hernia, carpal tunnel release, arthroscopy of the knee, Bell’s palsy, and all-cause mortality, did not differ significantly between cohorts, supporting the robustness of the matched cohorts and suggesting minimal residual confounding.

### Sensitivity Analyses

Four sensitivity analyses were conducted to evaluate the robustness of the primary findings. We repeated the analysis without the requirement for a biopsy before the index diagnosis in order to increase the number of eligible cases, thereby improving statistical power. This analysis was performed both with and without exclusion of patients with prior UM. In an additional sensitivity analysis, patients with CM were compared to an alternative control cohort consisting of individuals with a recorded encounter for screening for malignant neoplasm of the skin (ICD-10 Z12.83) or actinic keratosis (ICD-10 L57.0) to assess whether the observed associations were consistent across a different comparison group. This analysis was likewise performed both with and without exclusion of patients with prior UM. All four sub-analyses demonstrated a large positive association between CM and UM.

The findings were as follows:

- In an analysis in which the requirement for a biopsy before the index diagnosis was removed and we excluded UM diagnosis prior to CM, there were 360,611 patients in the CM group and 788,090 patients in the KC group. After PSM there were 333,110 patients in each group. During the follow-up period, UM occurred in 158 patients with CM compared with 12 patients with KC (0.05% vs. 0.004%, respectively). The corresponding HR remained markedly elevated (HR 14.32; 95% CI [7.96, 25.76]).
- In an analysis in which the requirement for a biopsy before the index diagnosis was removed and we did not exclude UM diagnosis prior to CM, there were 364,811 patients in the CM group and 811,468 patients in the KC group. After PSM there were 337,227 patients in each group. During the follow-up period, UM occurred in 1155 patients with CM (0.34%) compared with 36 patients with KC (0.01%), corresponding to an OR of 32.55 (95% CI [23.28, 45.53]; *p* < 0.0001).
- In an additional sensitivity analysis, comparing CM patients to the alternative control cohort and excluding UM diagnosis prior to CM, there were 371,512 patients in the CM group and 2,244,763 patients in the control group. After PSM there were 347,469 patients in each group. During the follow-up period, UM occurred in 169 patients with cutaneous melanoma compared with 18 patients in the alternative control cohort (0.049% vs. 0.005%, respectively). The corresponding hazard ratio remained high (HR 10.53; 95% CI [6.47, 17.11]).
- In the same additional sensitivity analysis, comparing CM patients with an alternative control cohort without exclusion of patients with UM diagnosis prior to CM diagnosis, there were 366,527 patients in the CM group and 2,222,880 patients in the control group. After PSM there were 343,399 patients in each group. During the follow-up period, UM occurred in 1177 patients with CM (0.34%) compared with 31 patients in the alternative control cohort (0.009%). This corresponded to an OR of 38.09 (95% CI [26.67, 54.42]; *p* < 0.0001).

Supplementary Figure S1 displays the Kaplan–Meier survival curves and corresponding forest plots for the incident UM risk.

## 4. Discussion

This study used the TriNetX global collaborative network to retrospectively examine the long-term risk of uveal melanoma in patients with cutaneous melanoma compared to those with keratinocyte carcinomas.

Previous studies examining the differences and similarities between CM and UM demonstrated similarities, including an increased risk in fair-skinned and light-colored-eyed individuals [16]. They differ in routes of metastatic spread, response to immunotherapy and survival chances. There is also a rise in worldwide frequency of CM, while UM incidence is stable [17].

As for mutation analysis, CM is characterized by frequent *BRAF* (V600), *NRAS*, and *NF1* mutations, high tumor mutational burden, and ultraviolet exposure-related changes, often linked to germline *CDKN2A* and low-penetrance *MC1R* variants [1,10,20,21]. In contrast, UM typically harbors mutually exclusive *GNAQ* or *GNA11* mutations initiating oncogenesis, with progression determined by *BAP1* loss (high metastatic risk), *SF3B1* mutations (intermediate risk), or *EIF1AX* mutations (low risk). UM also shows characteristic chromosomal alterations, including monosomy 3, 8q gain, and 6p gain, and overall low tumor mutational burden [7,22,23].

UV radiation is an important well-documented risk factor for CM. The current consensus is that UV exposure plays a limited and site-specific role in UM. Nonetheless, the selection of keratinocyte carcinoma (KC) patients as a control group was done to reduce confounding factors from different sunlight exposure patterns and access to healthcare between cohorts. Although KC is a biologically distinct malignancy from CM, it was retained as the primary comparator because patients with KC share important characteristics with patients with CM, including cumulative UV exposure, dermatologic surveillance, and healthcare-seeking patterns. The consistent association observed in sensitivity analyses using skin cancer screening and actinic keratosis cohorts further supports the robustness of the finding. Nevertheless, these alternative comparators cannot fully eliminate the possibility of residual biological or genetic confounding, particularly given the distinct molecular profiles of CM and KC. Therefore, our findings should be interpreted with caution.

Our study results demonstrate an elevated risk of UM among patients with CM compared to KC patients. Although the absolute number of UM events was small, reflecting the rarity of this malignancy, the association remained statistically significant after rigorous matching across comorbidities, ocular conditions, and healthcare utilization factors. These findings suggest a potential link between CM and UM, although they should be interpreted with caution given the limited number of observed events.

An important consideration is the potential for surveillance bias, as patients with CM might be expected to undergo more frequent ophthalmologic evaluations, increasing the likelihood of detecting UM. However, our control analysis demonstrated that ophthalmologic follow-up was slightly less frequent among patients with CM than among those with KC (OR 0.91; 95% CI [0.85, 0.97]). Therefore, the observed increase in UM incidence is unlikely to be explained by greater ophthalmologic surveillance and instead supports the presence of a true difference in risk between the cohorts. Our findings correlate with the findings of a study by Van Hees et al. from 1994, who found that more atypical nevi were found in UM patients than in age- and sex-adjusted controls (odds ratio of 2.9 for 1–2 atypical nevi versus none; odds ratio of 5.1 for >3 atypical nevi versus none) [24]. There was one previous population study comparing the incidence of CM and UM, partially corroborating our results. It was published in 2002 based on the Surveillance, Epidemiology and End Results (SEER) program from 1973 to 1998. It found that patients with an initial UM developed CM 4.6 times (95% CI 2.9–6.8) more often than the general population. In contrast, patients with initial CM were not subsequently diagnosed with UM at an appreciably elevated rate (standardized incidence ratio 1.4; 95% CI 0.5–3.0) [18]. Our findings show that UM preceded CM in 70% of the patients with both diagnoses, consistent with the directionality reported by Shors et al. However, our incident-UM sensitivity analysis showed that the association persisted after excluding patients with UM documented before CM (HR 14.32; 95% CI 7.96–25.76). Differences in study populations, ascertainment, study periods, and comparator definitions may account for the discrepancy with Shors et al. Given the rarity of UM and the small number of temporally ordered events, these findings should be interpreted cautiously and do not establish causality.

The strengths of our study include the large sample size, multicenter nature of the dataset, and careful matching to control for confounding factors. Limitations include reliance on ICD-10 codes without histopathologic confirmation, lack of direct UV exposure data, absence of genetic information, and inability to account for tumor stage or treatment. Additionally, we were unable to perform stratified analyses according to the World Health Organization (WHO) classification of cutaneous melanoma subtypes because the number of patients within individual subgroups was insufficient for meaningful analysis. This limitation was further compounded by the TriNetX privacy policy, which suppresses results for subgroups with fewer than 10 patients experiencing the outcome, preventing reliable reporting of these subgroup analyses. Another limitation of the TriNetX platform is the heterogeneity of the 150 healthcare centers included in the network, particularly regarding access to specialized ocular oncology diagnosis, which could differentially affect UM ascertainment. Nevertheless, this is also an advantage, providing us with diverse real-world data. Also, malignant neoplasm of the iris was not included since there is no matching ICD-10 code, while non-melanoma malignant neoplasms of the choroid and ciliary body were included, but those are immensely rare. The only non-melanoma malignant neoplasms of the choroid and ciliary body that could potentially affect our results were metastatic cutaneous melanoma to the choroid. To avoid this misclassification, we excluded patients diagnosed with metastatic disease. That meant patients with metastatic choroidal melanoma were also excluded, but we expect this would affect both cohorts similarly. Also, we saw that about two-thirds of the patients who had both UM and CM developed the UM prior to CM diagnosis. Since metastatic CM to the choroid is a late outcome, those patients had primary UM with high certainty.

## 5. Conclusions

Cutaneous melanoma is associated with a significantly higher risk of uveal melanoma compared to KC. UM diagnosis preceded CM in most patients with both diagnoses. However, in an incident-UM sensitivity analysis, the association persisted after excluding patients with UM documented before CM. These findings suggest a shared susceptibility between cutaneous and uveal melanoma of unknown origin that is not described in the literature at this point. It is only partially explained by extremely rare genetic predisposition syndromes such as *BAP1* tumor predisposition syndrome, which involves only 1–2% of UM patients, and dysplastic nevus syndrome. Note that this paper does not establish a causal link between CM and UM, merely an association.

Given the poor prognosis of metastatic UM, identification of high-risk groups is clinically important. While routine UM screening is not currently recommended for the general population, our findings suggest that clinicians managing patients with a history of CM should maintain heightened vigilance for ocular symptoms and consider ophthalmic examination, specifically with existing family history or atypical nevi [19,24]. On the other hand, Ophthalmologists following patients diagnosed with UM may consider a referral for dermatologic examination. Nevertheless, given the low absolute incidence of UM and the retrospective nature of the study, these findings should be considered hypothesis-generating and require confirmation in future studies. Further research is warranted to explore underlying mechanisms and guide risk-stratified screening strategies.

## Figures and Tables

**Table 1:** Baseline characteristics of patients with cutaneous melanoma and those with keratinocyte carcinomas over a 5-year follow-up. Data are presented for both the pre- and post-propensity score matching (PSM) cohorts. Abbreviations: SD, standard deviation; Std. diff., standardized difference; NA, not applicable.

Characteristic Name	Before PSM				After PSM			
	Cutaneous Melanoma (n = 57,352)	Keratinocyte Carcinoma (n = 137,083)	*p*	Std diff.	Cutaneous Melanoma (n = 52,860)	Keratinocyte Carcinoma (n = 52,860)	*p*	Std diff.
Age at index (mean ± SD)	61.39 ± 15.32	68.8 ± 12.43	<0.001	0.53	63.28 ± 13.76	63.52 ± 13.4	0.004	0.02
Female (%)	29,119 (51.57)	64,089 (46.75)	<0.001	0.10	26,530 (50.19)	26,178 (49.52)	0.0304	0.01
Male (%)	27,340 (48.42)	72,987 (53.24)	<0.001	0.10	26,326 (49.8)	26,680 (50.47)	0.0294	0.01
White (%)	52,285 (92.6)	128,091 (93.44)	<0.001	0.03	49,008 (92.71)	48,982 (92.66)	0.7587	0.00
Black or African American (%)	201 (0.36)	392 (0.29)	0.0113	0.01	186 (0.35)	173 (0.33)	0.4919	0.00
Hispanic or Latino (%)	610 (1.08)	1571 (1.15)	0.2135	0.01	568 (1.08)	511 (0.97)	0.0811	0.01
Overweight and obesity (%)	4046 (7.17)	11,679 (8.52)	<0.001	0.05	3961 (7.49)	3723 (7.04)	0.0048	0.02
Nicotine dependence (%)	1559 (2.76)	5899 (4.3)	<0.001	0.08	1545 (2.92)	1400 (2.65)	0.0067	0.02
Factors influencing health status and contact with health services (%)	32,786 (58.07)	100,068 (73.0)	<0.001	0.32	32,042 (60.62)	31,377 (59.36)	<0.001	0.03
Hypertensive diseases (%)	14,443 (25.58)	51,448 (37.53)	<0.001	0.26	14,338 (27.12)	14,034 (26.55)	0.0349	0.01
Disorders of lipoprotein metabolism and other lipidemias (%)	13,277 (23.52)	50,019 (36.49)	<0.001	0.29	13,240 (25.05)	12,913 (24.43)	0.0198	0.01
Diabetes mellitus (%)	4790 (8.48)	16,976 (12.38)	<0.001	0.13	4757 (9.0)	4573 (8.65)	0.046	0.01
Chronic kidney disease (%)	4701 (8.33)	21,994 (16.04)	<0.001	0.24	4685 (8.86)	4225 (7.99)	<0.001	0.03
Diseases of the eye and adnexa (%)	2118 (3.75)	11,831 (8.63)	<0.001	0.20	2115 (4.0)	2197 (4.16)	0.2023	0.01
Age-related cataract (%)	1503 (2.66)	7424 (5.42)	<0.001	0.14	1503 (2.84)	1354 (2.56)	0.0047	0.02
Glaucoma (%)	924 (1.64)	4613 (3.36)	<0.001	0.11	923 (1.75)	823 (1.56)	0.0158	0.01
Other retinal disorders (%)	924 (1.64)	5214 (3.8)	<0.001	0.13	923 (1.75)	835 (1.58)	0.0343	0.01
Visit: Ambulatory (%)	48,575 (86.03)	125,299 (91.4)	<0.001	0.17	45,993 (87.01)	45,933 (86.9)	0.5838	0.00
Visit: Emergency (%)	3877 (6.87)	16,203 (11.82)	<0.001	0.17	3822 (7.23)	3678 (6.96)	0.0845	0.01
Encounter for examination of eyes and vision (%)	110 (0.2)	486 (0.36)	<0.001	0.03	109 (0.21)	73 (0.14)	0.0076	0.02
Ophthalmological services: medical examination and evaluation, with initiation or continuation of diagnostic and treatment program; comprehensive, established patient, 1 or more visits (%)	1268 (2.25)	6364 (4.64)	<0.001	0.13	1267 (2.4)	1133 (2.14)	0.0057	0.02
Ophthalmological services: medical examination and evaluation, with initiation or continuation of diagnostic and treatment program; intermediate, established patient (%)	351 (0.62)	1472 (1.07)	<0.001	0.05	349 (0.66)	316 (0.6)	0.1992	0.01
Ophthalmological services: medical examination and evaluation with initiation of diagnostic and treatment program; comprehensive, new patient, 1 or more visits (%)	274 (0.48)	1446 (1.06)	<0.001	0.07	274 (0.52)	206 (0.39)	0.0019	0.02
Ophthalmological services: medical examination and evaluation with initiation of diagnostic and treatment program; intermediate, new patient (%)	32 (0.06)	123 (0.09)	0.0195	0.01	32 (0.06)	24 (0.04)	0.2849	0.01
Chemotherapy (%)	2711 (4.8)	14,276 (10.41)	<0.001	0.21	2658 (5.03)	2699 (5.11)	0.5653	0.00
Corticosteroids (%)	8917 (15.79)	33,204 (24.22)	<0.001	0.21	8786 (16.62)	8570 (16.21)	0.0729	0.01
Antimetabolites (%)	1176 (2.08)	9331 (6.81)	<0.001	0.23	1176 (2.22)	1270 (2.4)	0.0545	0.01
Alkylating agents (%)	104 (0.18)	319 (0.23)	0.0378	0.01	103 (0.2)	78 (0.15)	0.0629	0.01

**Table 2:** Summary of odds ratios (ORs) with 95% confidence intervals (95% CIs) and corresponding Wald test *p*-values for malignant neoplasms of the choroid or ciliary body in patients with cutaneous melanoma and those with keratinocyte carcinomas, over a 5-year follow-up period.

Outcome	Patients in Cohort		Patients with Outcome		Incident Risk (%)		OR [95% CI]	*p*-Value
	Cutaneous Melanoma	Keratinocyte Carcinoma	Cutaneous Melanoma	Keratinocyte Carcinoma	Cutaneous Melanoma	Keratinocyte Carcinoma		
Malignant neoplasm of choroid or ciliary body	52,860	52,860	53	13	0.10	0.02	4.08 [2.22, 7.49]	<0.001
Ophthalmologic follow-up	52,860	52,860	1646	1805	3.11	3.41	0.91 [0.85, 0.97]	0.005
Appendicitis	52,683	52,617	94	102	0.18	0.19	0.92 [0.69, 1.21]	0.53
Inguinal hernia	51,561	51,253	687	676	1.33	1.32	1.01 [0.91, 1.13]	0.84
Carpal tunnel release	52,558	52,514	220	220	0.42	0.42	1.00 [0.83, 1.21]	1
Arthroscopy of knee	52,575	52,490	100	114	0.19	0.22	0.88 [0.67, 1.15]	0.34
Bell’s palsy	52,661	52,614	81	101	0.15	0.19	0.80 [0.60, 1.08]	0.14
Deceased	52,734	52,713	2120	2217	4.02	4.21	0.95 [0.90, 1.01]	0.13

## Data Availability

The data can be accessed on the website https://trinetx.com accessed on 17 June 2026.

## Supplementary Materials

The following supporting information can be downloaded at https://www.mdpi.com/article/10.3390/cancers18172826/s1, Figure S1. Association between cutaneous melanoma and incident uveal melanoma in sensitivity analyses. (A) Forest plot demonstrating hazard ratios (HRs) with 95% confidence intervals (95% CIs) for the risk of malignant neoplasms of the choroid or ciliary body among patients with cutaneous melanoma compared with the corresponding control cohorts. (B,C) Kaplan-Meier survival curves demonstrating time to malignant neoplasms of the choroid or ciliary body among patients with cutaneous melanoma and control cohorts in the first and second sensitivity analyses, respectively. Abbreviations: CM, cutaneous melanoma; KC, keratinocyte carcinoma; HR, hazard ratio; CI, confidence interval; UM, uveal melanoma.

## References

[1] Jager M.J., Shields C.L., Cebulla C.M., Abdel-Rahman M.H., Grossniklaus H.E., Stern M.H., Carvajal R.D., Belfort R.N., Jia R., Shields J.A. (2020). Uveal melanoma. Nat. Rev. Dis. Primers.

[2] Kaliki S., Shields C.L. (2017). Uveal melanoma: Relatively rare but deadly cancer. Eye.

[3] Chattopadhyay C., Kim D.W., Gombos D.S., Oba J., Qin Y., Williams M.D., Esmaeli B., Grimm E.A., Wargo J.A., Woodman S.E. (2016). Uveal melanoma: From diagnosis to treatment and the science in between. Cancer.

[4] Ortega M.A., Fraile-Martínez O., García-Honduvilla N., Coca S., Álvarez-Mon M., Buján J., Teus M.A. (2020). Update on uveal melanoma: Translational research from biology to clinical practice (Review). Int. J. Oncol..

[5] Bai H., Bosch J.J., Heindl L.M. (2023). Current management of uveal melanoma: A review. Clin. Exp. Ophthalmol..

[6] Wespiser M., Neidhardt E., Negrier S. (2023). Uveal melanoma: In the era of new treatments. Cancer Treat. Rev..

[7] Fallico M., Raciti G., Longo A., Reibaldi M., Bonfiglio V., Russo A., Caltabiano R., Gattuso G., Falzone L., Avitabile T. (2021). Current molecular and clinical insights into uveal melanoma (Review). Int. J. Oncol..

[8] Souto E.B., Zielinska A., Luis M., Carbone C., Martins-Gomes C., Souto S.B., Silve A.M. (2019). Uveal melanoma: Physiopathology and new in situ-specific therapies. Cancer Chemother. Pharmacol..

[9] Cao L., Chen S., Sun R., Ashby C.R., Wei L., Huang Z., Chen Z.S. (2023). Darovasertib, a novel treatment for metastatic uveal melanoma. Front. Pharmacol..

[10] Gosman L.M., Țăpoi D.A., Costache M. (2023). Cutaneous Melanoma: A Review of Multifactorial Pathogenesis, Immunohistochemistry, and Emerging Biomarkers for Early Detection and Management. Int. J. Mol. Sci..

[11] Gandini S., Sera F., Cattaruzza M.S., Pasquini P., Zanetti R., Masini C., Boyle P., Melchi C.F. (2005). Meta-analysis of risk factors for cutaneous melanoma: III. Family history, actinic damage and phenotypic factors. Eur. J. Cancer.

[12] Luke J.J., Flaherty K.T., Ribas A., Long G.V. (2017). Targeted agents and immunotherapies: Optimizing outcomes in melanoma. Nat. Rev. Clin. Oncol..

[13] Robert C., Schachter J., Long G.V., Arance A., Grob J.J., Mortier L., Daud A., Carlino M.S., McNeil C., Lotem M. (2015). Pembrolizumab versus Ipilimumab in Advanced Melanoma. N. Engl. J. Med..

[14] Gershenwald J.E., Scolyer R.A., Hess K.R., Sondak V.K., Long G.V., Ross M.I., Lazar A.J., Faries M.B., Kirkwood J.M., McArthur G.A. (2017). Melanoma staging: Evidence-based changes in the American Joint Committee on Cancer eighth edition cancer staging manual. CA Cancer J. Clin..

[15] Chang A.E., Karnell L.H., Menck H.R. (1998). The National Cancer Data Base report on cutaneous and noncutaneous melanoma: A summary of 84,836 cases from the past decade. The American College of Surgeons Commission on Cancer and the American Cancer Society. Cancer.

[16] Brănișteanu D.E., Porumb-Andrese E., Stărică A., Munteanu A.C., Toader M.P., Zemba M., Porumb V., Cozmin M., Moraru A.D., Nicolescu A.C. (2023). Differences and Similarities in Epidemiology and Risk Factors for Cutaneous and Uveal Melanoma. Medicina.

[17] Pandiani C., Béranger G.E., Leclerc J., Ballotti R., Bertolotto C. (2017). Focus on cutaneous and uveal melanoma specificities. Genes Dev..

[18] Shors A.R., Iwamoto S., Doody D.R., Weiss N.S. (2002). Relationship of uveal and cutaneous malignant melanoma in persons with multiple primary tumors. Int. J. Cancer.

[19] Smith J.H., Padnick-Silver L., Newlin A., Rhodes K., Rubinstein W.S. (2007). Genetic study of familial uveal melanoma: Association of uveal and cutaneous melanoma with cutaneous and ocular nevi. Ophthalmology.

[20] Newton-Bishop J., Bishop D.T., Harland M. (2020). Melanoma Genomics. Acta Derm. Venereol..

[21] Johansson P.A., Brooks K., Newell F., Palmer J.M., Wilmott J.S., Pritchard A.L., Broit N., Wood S., Carlino M.S., Leonard C. (2020). Whole genome landscapes of uveal melanoma show an ultraviolet radiation signature in iris tumours. Nat. Commun..

[22] Smit K.N., Jager M.J., de Klein A., Kiliҫ E. (2020). Uveal melanoma: Towards a molecular understanding. Prog. Retin. Eye Res..

[23] Reggiani F., Ambrosio M., Croce M., Tanda E.T., Spagnolo F., Raposio E., Petito M., Rashed Z.E., Forlani A., Pfeffer U. (2023). Interdependence of Molecular Lesions that Drive Uveal Melanoma Metastasis. Int. J. Mol. Sci..

[24] van Hees C.L., de Boer A., Jager M.J., Bleeker J.C., Kakebeeke H.M., Crijns M.B., Vandenbroucke J.P., Bergman W. (1994). Are atypical nevi a risk factor for uveal melanoma? A case-control study. J. Investig. Dermatol..

