# Supplementary material for: A Possible Link Between Cutaneous Melanoma and Uveal Melanoma: A Multicenter Retrospective Cohort Study
Source: Cancers (Basel). 2026 Sep 1;18(17):2826. doi: 10.3390/cancers18172826 (PMC13564481; doi:10.3390/cancers18172826)
Supplement: Supplementary file 1 [file cancers-18-02826-s001.zip › cancers-4440860-supplementary.pdf]

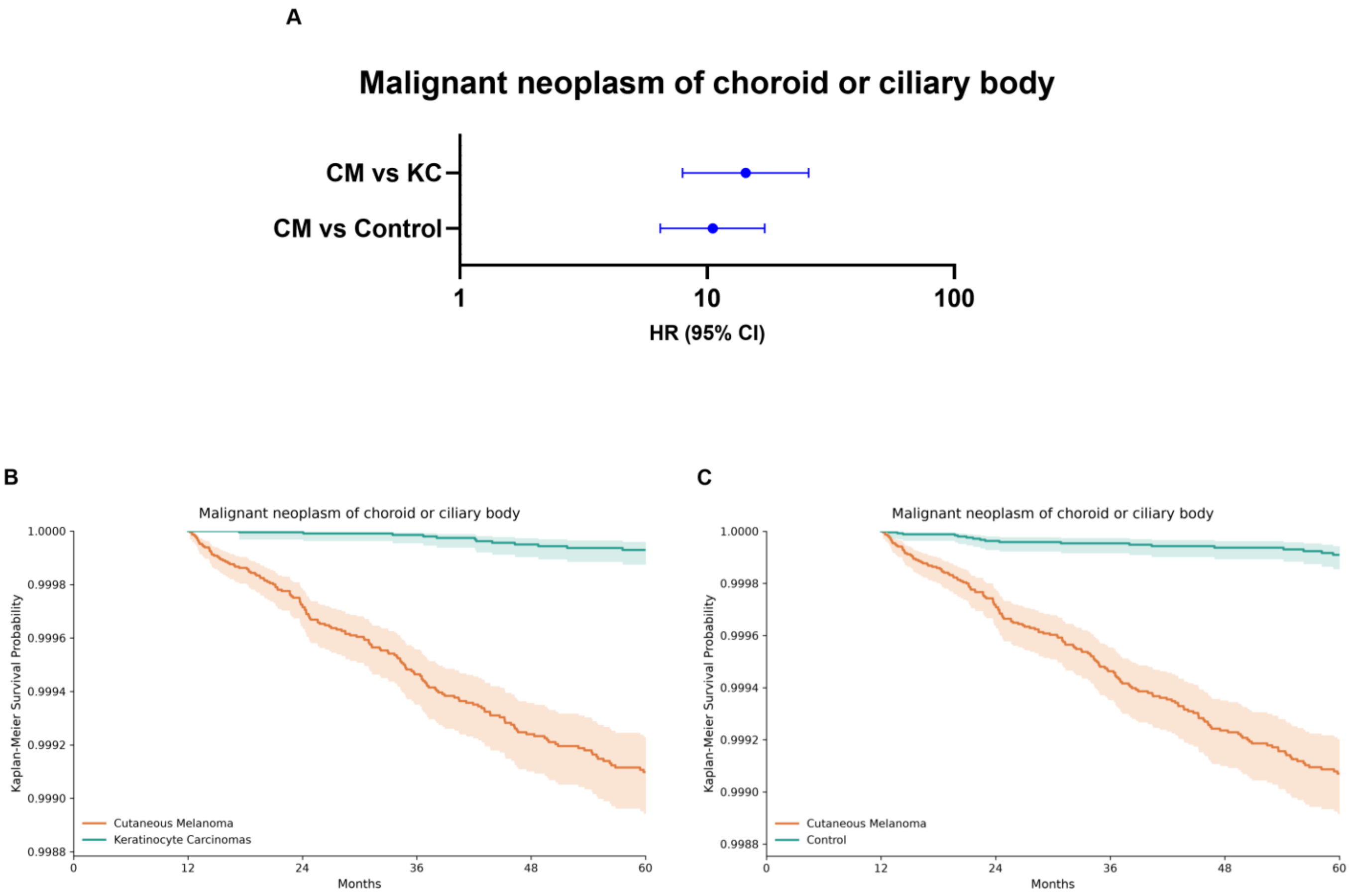

Supplementary Figure S1. Association between cutaneous melanoma and incident uveal melanoma in sensitivity analyses. (A) Forest plot demonstrating hazard ratios (HRs) with 95% confidence intervals (95% CIs) for the risk of malignant neoplasms of the choroid or ciliary body among patients with cutaneous melanoma compared with the corresponding control cohorts. (B-C) Kaplan-Meier survival curves demonstrating time to malignant neoplasms of the choroid or ciliary body among patients with cutaneous melanoma and control cohorts in the first and second sensitivity analyses, respectively. Abbreviations: CM, cutaneous melanoma; KC, keratinocyte carcinoma; HR, hazard ratio; CI, confidence interval; UM, uveal melanoma.
